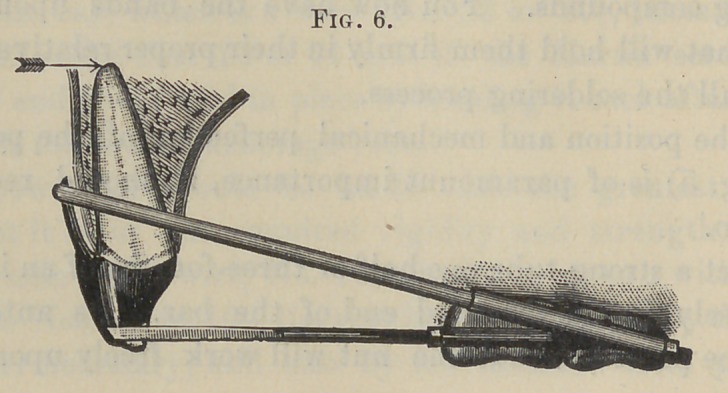# The Aesthetic Correction of Facial Contours in the Practice of Dental Orthopedia

**Published:** 1895-11

**Authors:** C. S. Case


### The Æsthetic Correction of Facial Contours in the Practice of Dental Orthopedia.

BY C. S. CASE, M.D., D.D.S.

Read before the Tri-State Meeting at Detroit, June 18th, 1895.

I use the term "dental orthopedia" in preference to that of orthodontia because it is more applicable to our present advancement in this department of dentistry. The latter word, being derived from the two Greek words *orthos*, straight, and *odous*, tooth, is confined to the straightening or regulating of the teeth; whereas the present definition of orthopedia—from *orthos*, straight, and *pais*, child—is "the surgical and mechanical correction of deformities of children and of deformities in general." The prefix "dental" localizes its scope to the region of the teeth. Its meaning now may be confined to straightening the position of the teeth, and also to the correction of all deformities that are due to a malposition of the teeth or that can be corrected through the medium of the teeth.

The correction of certain deformities of the face, or the æsthetic remodeling of the natural features by changing the shape and surface contour of the bone over that region of the face that can be affected by force appliances attached to the teeth, has been the subject of numerous papers presented by me before leading dental societies. And as these have been widely published, I presume that the majority of my present hearers are well informed in regard to my claims and the principles, at least, involved in the treatment I have proposed.

The principal object of presenting the subject at this meeting is to afford an opportunity to many who have been unable to see cases I have treated, or personally examine the plaster casts, which, I have reason to believe, speak far more eloquently of the truthfulness of what I have been able to accomplish in this class of dento-facial irregularities than it is possible for me to express in words.

Of these casts, which I here present, the larger portion have been selected from cases that were used to illustrate the papers read at the World's Columbian Dental Congress and the last March meeting of the Odontological Society of New York City, and published in the proceedings of these meetings. In addition, I now present the casts of three cases that have not been shown elsewhere (see cases 3, 4, 5), the whole comprising every variety of that class of facial deformities that are due to a marked irregularity of the teeth accompanied by a facial defect that can be remedied only by a decided movement of the roots of the anterior superior teeth. This does not refer to the far more common deformities that are caused by a malposition of the crowns of the anterior superior teeth, nor does it include those very common forms of facial imperfections where, though the teeth themselves show no special irregularity, they can be used, without harm to their usefulness or position, as means for applying force that will result in a decided beautifying of the face.

I believe the time is not far distant when the skillful operator in dental orthopedia will be able to æsthetically correct and beautify many common types of facial imperfection which we ordinarily recognize and denominate as "plain," "unattractive," and even "ugly."

This will be accomplished by force appliances attached to the teeth and worn for a few months during youth, or at a time when the immaturity of the bones permits them to yield most readily to the proper force, the teeth in these instances subserving the purpose of convenient places for attaching the appliances, and, through this medium, of directing and applying the force to the bones over that portion of the face which requires movement.

This subject is a new one, and, as Dr. Farrar has kindly remarked, "decidedly an advance step in orthodontia." It, moreover, pertains to a practice somewhat beyond the scope of dentistry proper, and yet one that cannot be touched by the general orthopedic surgeon, because it requires for its perfect accomplishment an intimate knowledge of the teeth and the manipulative skill of a dentist. I therefore present it to you for what it is worth, with the belief that those who care to give the subject any thought must be convinced of its possibilities after a careful examination of these models, leaving it for those who have been intimately associated with some of the cases I have treated to convince you of its practicability.

A second object of this paper will be to modify some of the sanguine expressions in my early publications on the subject.

In the first half-dozen cases of this character which I treated, I was fortunate in meeting with no obstacle whatever. In fact, the very first case for which I invented the peculiar apparatus, which I have used so successfully for applying force to the roots of the teeth responded so readily in every respect that I was able to make a greater change in the positions of the teeth and shape of the face than I have since been called upon to accomplish.

See Case 1, Miss S., age 13. By examining the plaster casts which were made at the beginning of the operation, it will be found that the upper dental arch was decidedly small and retracted, while the lower was large and prominent, with the peculiar open occlusion characteristic of a mouth-breather.

This gave to the face a long, narrow, and decidedly angular appearance. The lower lip protruded with an entire obliteration of its usual graceful curve, while the upper lip and middle features

CASE 1.

of the face were equally depressed. The lower part of the nose being drawn back by its muscular attachment to the depressed bone, assumed a thin and pinched appearance. Within seven months from the commencement of treatment, much to my surprise and the pleasure of all, this was corrected, and to-day, as has often been remarked, she is quite a handsome young lady, the final model of her face hardly doing justice to her present appearance.

This and similar successes led me to the conclusion and published opinion that I could with perfect facility move the anterior portion of the superior maxillary process forward or back to any extent that a case might demand; and also to the statement that when force was applied to the anterior teeth in phalanx in the manner described, they would not move by virtue of the absorption of the walls of the sockets, as ordinarily occurs, but that the entire bony structure in which they were imbedded would be carried bodily forward or back with the roots.

I now desire to say that I believe my original claims will hold good in nearly all cases where the treatment is attempted sufficiently early in life, but that instances will occasionally arise when the contrary seems to be true.

When abnormal prominence of the features occurs along the upper portion of the superior lip and lower portions of the nose, caused by the position of the roots of the anterior superior teeth and maxillary process, reduction can rarely be performed with ease. Especially is this true if caused by the anterior position of the roots of the cuspids. These roots being surrounded by the most massive and dense part of the superior maxillæ, in which they are deeply imbedded, together with the fact that their position is such that their movement bodily in a posterior direction necessarily requires the absorption of a large portion of bone, makes this operation one of the most difficult in dental orthopedia.

The posterior movement of the incisor roots is not as difficult, as can be well understood by examining a macerated skull. The bone in this region, because of its peculiar shape, will usually respond to the proper force by bending bodily.

Especially is this true when force is applied in the anterior direction, as is well shown by the casts of a number of the cases which I have brought for your inspection. And while this movement in a posterior direction would theoretically seem to be impossible for an adult on account of the position and early ossification of the vomer, I am pleased to call your attention particularly to the practical demonstration of this principle for patients older than eighteen.

Case 2 is that of a young lady who was twenty years of age when I commenced treatment.

It will be seen by an examination of the models that the roots of the anterior teeth, at the beginning of the operation, were very prominent. The crowns of the incisors being inclined inward, gave to the face a bulged and very unhappy expression along the superior portion of the upper lip, affecting the shape of the nose. You will see the same expression in the beginning face model of Case 3. This condition, more or less intensified, is not an uncommon one. If you have not often observed it among your patients and others, it is because you have not learned to classify it among the conditions which demand your skill.

The teeth of these persons are often in proper alignment, and through long habit and avocation you proceed to treat them with

Case 2.

little heed to the facial defect with their position produces. If you think of it at all, it is to become conscious that the face is homely, plain, or ugly—made that way—unchangeable.

And so the subject is dismissed as one which makes no appeal to you; yet this, as well as many other facial imperfections that are produced by a malposition of the teeth, is a condition which, if taken early, can be remedied with no great difficulty; and in doing so you will confer one of the greatest of human blessings. Now see what I have accomplished for this young lady. Notice in the final model of her face the improvement of the shape of her nose, the ease and graceful curve of her upper lip, and the natural pose and perfection of her mouth. To-day she possesses a face of more than passing beauty, produced from one that was, to say the least, exceedingly plain and unattractive. Nor is it possible for you, as in other cases, to fully appreciate the change by these models, the difference in all these cases being far more marked in conversation.

But what of the difficulties? Please to remember that a person over twenty years of age is not a typical case for moving the roots of the six anterior teeth in a posterior direction.

The wonder is that I was able to accomplish so much. The first bicuspids were extracted. The second bicuspid and mo-

Fig. 1.

Fig. 2.

lars on each side were banded, and the bands firmly united and cemented to the teeth. From these anchorage attachments the reciprocating force bars extended to the anterior teeth, as will be described later. For nearly one year the great power of this apparatus was continually exerted in forcing the roots of the six anterior teeth and their surrounding bone to take a more posterior position. During this time the spaces produced by the extraction of the bicuspids were entirely closed; and, as a proof of the amount of force that was used, this was accomplished almost wholly by a forward movement of the posterior teeth; not by tipping their crowns forward, but by bodily moving them almost in an upright position through the process. For, as will be observed by a careful study of the models, the anterior teeth at their cervices have been forced back but very little. The occluding ends of the teeth, especially of the incisors, have been forced slightly forward. The great force being exerted at their roots seems to have moved the apices back at least one-eighth of an inch. Fig. 1 fairly represents the original relative position of the central incisors during a masticating occlusion, and Fig. 2 the present position.

Case 3 is that of a young man who was eighteen years of age when I commenced treatment a little over one year ago. The

Case 3.

teeth are large and strong, jaws and bones proportionately large and rigid. The facial prominence or bulging of the face in the region of the wings of the nose was far more pronounced than was the former case, and unfortunately due largely to the anterior position of the roots of the cuspids. After the first bicuspids were extracted, anchorage attachments were made for the posterior teeth as before, but the reciprocating force bars extended only to the cuspids. The anchorage was further enforced in this case by rubber bands extending from the upper attachment of the cuspids to the posterior ends of a bar that was attached to all of the lower teeth, as has been repeatedly described by me elsewhere.

The power of this apparatus was continued from May, 1894, for eight months, since which time to June 10, 1895, the incisors

Case 4.

have been included by an extension of the force bars. I removed the apparatus at this time for the purpose of taking impressions to bring the models of the case before you in its present incomplete state.

The bicuspid spaces have been closed partly by the forward movement of the anchorage teeth, but not so much as in the former case, nor have I been able to retain them in as upright a position. The roots of the cuspids, which seemed to present an almost insurmountable resistance to far more force than I have ever employed in any other case, have moved appreciably, but not as much as I hoped. I leave it for you to judge of the improvement, which is quite marked in some particulars, not shown by the face models, and due to the regulation of the incisors.

Now, I wish to introduce to your notice two cases, 4 and 5, which are the only ones where I have attempted a forward movement of the roots of the superior incisors, that the surrounding process and immediately adjoining bone did not move bodily forward with the teeth.

Case 4, Miss J., aged sixteen, commenced treatment September 12, 1894.

Case 5, Miss F., aged fourteen, commenced treatment September 22, 1894.

In both these cases, as soon as there was an appreciable movement it was accompanied by a decided prominence over each root, showing that the roots had moved by the immediate absorption of the sockets and the bending outward of the anterior alveolar plate. In one instance, before my attention was called to it, I could distinctly see the shape and position of the apices of some of the roots, which looked as though they were just ready to burst through the gum. Whenever this condition seemed to endanger the possibilities of success, the force was reduced, but not sufficient to allow the roots to return. Then I would wait for the ridges to be evened up by nature building in new tissue, when force would be again applied for a little further movement.

The necessity of these interruptions in the progress of the movement has required for these operations a much longer time than would otherwise have been necessary, and though neither are complete, the present results are quite satisfactory as regards the possibility of bringing about the desired position of the teeth.

Case 5.

Considered, however, from the standpoint of æsthetic facial development, they can never be as successful as they would have been had I been able to command a movement of a greater area of the superior maxillary bones. Had the teeth in many other cases I have treated moved in the same manner, they would have proven utter failures in the main object that was successfully attained, because they required something more than the mere movement of the roots and alveolar process. And this is true, though fortunately to a somewhat less extent, of these cases.

Before proceeding with the reading of the remainder of my paper, which pertains to the construction of apparatus, I will briefly describe the models of other cases I have brought for your examination, and which have been used to illustrate other papers presented at other meetings.

Case 6.

Case 6, Miss M., aged sixteen; commenced treatment December 26, 1893; staying bands October 15, 1894.

In this case the upper jaw was too small for the teeth, which were greatly crowded, and with the cuspids, as will be seen, in their customary positions under these conditions. The dental arch was lacking in its anterior extension rather than width, the incisors being quite posteriorly placed as regards the other teeth, producing a marked depression of the upper lip that was decidedly inharmomous, to say the least. In preparing it for the application of the contouring apparatus, the crowns of the incisors were first forced forward with jack-screws, and the cuspids crowded down more nearly into alignment. At this stage in the operation, models of the case were exhibited at the Illinois State Dental Society, to show the common facial result of the ordinary method of correcting this character of irregularity. The crowns of the incisors were pushed forward at a considerable angle, and all the teeth were crowded, with contracted interproximate spaces. The incisive fossæ seemed deeper than ever, while the facial imperfection was unimproved.

Now, mark the change which occurred after wearing this, the contouring apparatus, four months. Notice the upright position of the incisor, and the ample room that has been obtained for all the teeth; and, moreover, this change has produced, as in other instances, a decidedly favorable improvement in the face.

CASE 7.

Case 7 is that of a girl, thirteen years of age when I commenced treatment, and which was finished in six months.

I wish you to particularly examine this case, because, more than all the rest, it exemplifies the entire movement of the incisors and intermaxillary process, without the slightest apparent change in the position of any of the other teeth, with the exception of the cuspids, which were allowed to fall into more perfect alignment.

Case 8.

Case 8 is that of a Jewess, thirteen years of age, which was also finished in less than six months.

This was an inherited family type, and one that is often mistaken for a prognathous jaw, and occasionally treated with a submental splint and head-gear, in an attempt to force the chin back.

The superior maxilla was so small and retracted that the teeth flared outward to meet the lowers.

Treatment in this case consisted in expanding the dental arch forward and laterally, and so applying the force that there was a much greater movement of the roots than the crowns of the teeth. This resulted happily in a general enlargement of the maxillary process, with a much fuller contour to the middle features of the face, even to straightening the nose, as in Case 1.

Case 9 is that of a boy, fourteen years of age, which I will leave for Dr. Cushing to describe, as it is one of the cases he referred to me.

In answer to numerous inquiries which I receive, I have decided, in this connection, to describe and fully illustrate some of the important features of the latest methods I have adopted in the construction and application of the contouring apparatus.

I do this with the hope that some of the difficulties I encountered in my first cases may be avoided by you, and which were partly due to the comparatively crude construction and application of the apparatus I used then and published in my early writings upon this subject.

"The limited area upon which force can be applied to a tooth, compared to that portion covered by the gum and imbedded in a bony socket, has made it next to impossible, with all ordinary methods, to move the apex of the root in the direction of the applied force; nor could this ever be accomplished with force exerted in the usual way at one point upon the crown, however near the margin of the gum it be applied, for the opposing margin of the alveolar socket must receive the magnitude of this direct force, and in proportion to its resistance it will become a fulcrum, exerting a tendency to move the apex of the root in the opposite direction."

But if in the construction of the apparatus a static fulcrum is created, independent of the alveolus, at a point near the occluding portion of the crown, while the power is applied at a point as far upon the root as the mechanical and other opportunities of the case will permit, the apparatus becomes a lever of the third kind, the power being directed to a movement of the entire root in the direction of the applied force.

CASE 9.

This proposition is made plain by reference to the diagrams. In Fig. 3 let *A* be a point upon a central incisor at which force is applied in the direction indicated by the arrow, then will the

FIG. 3.

FIG. 4.

opposing wall, *B*, of the alveolar socket, near its margin, receive nearly all of the direct force, and in proportion to its resistance will there be a tendency to move the root in the opposite direction. This proposition will also hold good even if we apply the force at *A*, Fig. 4, or as far upon the root as may be permitted,

FIG. 5.

by attaching a rigid upright bar, *C*, to the anterior surface of the crown; the only difference being that we distribute the direct force over a greater area. But if, as in Fig. 5, we attach to the lower end of *C* a traction-wire or bar, *F*, and further enforce the mechanical principles of our machine by uniting its posterior attachment to the anchorage of the power bar *P*, we will have neutralized our anchorage force materially and created an independent static fulcrum at *D*. Our apparatus now will distribute its force over the entire root and give us complete direction and control of whatever power we put into it.

The entire tooth can be carried forward bodily, or either end can be made to move the more rapidly. The force thus directed to the ends of the roots will have an increased tendency to move the more or less yielding cartilaginous bone in which they are imbedded.

For material for regulating appliances I prefer German silver, not because of its inexpensiveness, but because much experience with all metals has taught me that none possess the same favorable qualities for this work.

The bands which surround the teeth should be wide and thin. If No. 10 Band S. G. wire is rolled to four or four and a half thousandths of an inch, it will usually be about the right width. This banding material should be drawn firmly around the natural teeth, the ends bent sharply to a right angle for the joint. When these are soldered the joint should project about a thirty-second of an inch, with its sharp corners clipped. Then the bands should be carefully fitted and burnished to the teeth with the joints a little to one side of the center of their anterior faces, to allow the upright bar to take its proper position, exactly in the center and parallel with the long axis of the tooth, and also to serve as a strengthening girder to the attachment. These and other small details may seem unnecessary, and yet, practically, they are of vital importance in the construction and application of the apparatus. It will be remembered that I originally made these upright bars of flattened No. 18 wire, leaving the ends long enough to bend over when in place and clasp the force bars. The operation of bending the bars was often a difficult and painful one, especially when it became necessary to remove and re-cement a band.

For upright bars I now cut pieces from Nos. 15 or 16 wire, about three-fourths of an inch long. These are filed slightly at the middle to receive the band, to which they are firmly soldered in the position described. Then they are bent and filed so as to fit perfectly the face of the tooth against which they are to rest. They should also follow the curve of the gum, nearly touching it, and extend above its free margin about one-fourth of an inch. The perfecting of these can only be accomplished at the chair.

Finally, the bar is shaped with a file according to whether force is to be applied in an anterior or a posterior direction.

I will first describe the method of procedure for cases which require a forward movement of the roots. In cases of this character I have never found it necessary to apply force to other roots than those of the incisors. The cuspids are usually retarded from taking their positions of alignment by the posterior position of the incisors, and are frequently so prominent that it first becomes necessary to force the crowns of the incisors forward with jack-screws or otherwise before the contouring apparatus can be effectively placed.

Usually in those conditions when the cuspids interfere, the upper ends of the upright bars can be at first ligated to the power bar, and thus the incisors forced forward until the power bar can be slipped into its proper position, which, as will be described, is always back of the upper end of the upright bars, against which it presses for the purpose of exerting force as high upon the roots to be moved as possible.

The posterior surface of that portion of the upright bars which stands in front of the gum is filed flat, so that their antero-posterior thickness tapers to one-half their original diameter at the ends, where they serve as rests for the power bar.

The anterior surfaces of the ends are rounded and polished to a thin edge. These ends should not extend above the upper edge of the power bar, unless it seems necessary to bend them at the extreme end to form a catch to prevent the power bar from sliding up.

The lower ends are grooved with a small round file to receive the fulcrum bar, which is a wire (No. 22 or 20), threaded only at one end in the Nos. 12 or 11 hole of the Martin screw-plate, the other end being held in place by bending it back after passing it through the lower anchorage tube.

The power bar should be made with the greatest care, in order that it be of the required rigidity and strength. Extra hard German silver wire, No. 10, should be drawn without annealing to Nos. 13 to 16—the size being regulated by the probable power necessary, and also by the distance from points of attachment and application. In other words, when the anterior end of the anchorage tube (*Pt*) at which the nut works is even with the bicuspids or at no great distance from the points of applying the force, less rigidity of the bar will be requisite; and again, for very young patients or where little power will be needed for the required movements. Ordinarily, however, No. 13 will not be found too large.

When it has been drawn to the proper size or selected and cut about the right length, that portion which is to extend between the right and left first bicuspids should be flattened in the rollers to about one-half its diameter. Then it should be bent so as to conform to the shape of the gum along the line where it is to rest. After bending closely over the cuspids, it should extend straight back into the tubes, into which its threaded ends should pass from one-half to three-fourths of an inch.

For more complete direction in the proper method of cutting a screw, making drills, taps, and nuts, I refer you to other writings where I have fully described the process.

The construction of the anchorage attachment, which now remains to be described, is of the greatest importance to the ease and accuracy of its application and subsequent usefulness.

Two molars, or the first molar and a bicuspid, and sometimes all three, should be selected for the anchorage teeth. When these are accurately fitted with wide bands, an impression in compound, of one side at a time, including the cuspids, should be taken. The bands should then be removed from the teeth without bending, and carefully placed in their proper position in the impression, which should be filled with Teague's or other investing compounds. You now have the bands upon a small model that will hold them firmly in their proper relative positions during all the soldering process.

As the position and mechanical perfection of the power tube (*Pt*, Fig. 5) is of paramount importance, it should receive first attention.

Select a strong tube one-half or three-fourths of an inch long, that loosely fits the threaded end of the bar. Its anterior end should be placed so that the nut will work freely upon the bar without impingement upon band, tooth, or gum, and it should take a direction that points exactly to that place upon the cuspid over which the power bar is to extend. In order to strictly observe this important direction, it usually becomes necessary to raise one or the other end of the tube from the bands by the intervention of lifts. It is often convenient to rest its posterior end upon the lever tube, its sharp projecting edges being rounded so as not to irritate the cheek.

The lever tube (*Ft*, Fig. 5) should also loosely fit its bar or wire, and be soldered directly to the bands, which it firmly unites, and thus serves to give statical strength to the anchorage. Their direction is not as material as that of the power tubes, because of the smallness and flexibility of the lever wire. Their posterior ends should project sufficiently free from the other parts to admit of the working of the nut. And in those instances where reciprocating rubber bands are to extend to a lower appliance—the advantage of which has been explained elsewhere—I allow these tubes to project for that purpose, finding them much more convenient than the buttons which I formerly used.

The tubes now being fitted with their joints turned toward the bands, they are attached with an abundance of silver solder, the bands also being united along their proximal surfaces.

All the parts which have undergone the soldering process are now boiled in sulphuric acid to remove the borax and oxid, after which the entire apparatus is polished and heavily gold-plated.

The teeth being properly separated with wax tape, the anchorage appliances should first be fitted to place in the mouth, and the cement allowed to harden before proceeding further, to pre-

FIG. 6.

vent dislodgment by the force necessary in placing the power-bar in position, especially as it often becomes necessary to remove and re-bend the bar several times in the final perfecting of its shape.

With the anchorage appliances and power-bar in place, the bands for the anterior teeth may now be fitted and cemented, allowing the upper ends of the upright bars to rest in front of the power-bar. Finally, the lever-bar is placed and the contouring apparatus is ready to commence the application of force at the next sitting.

An apparatus for moving the roots of the anterior teeth in a posterior direction is, in the main, constructed quite similarly. See Fig. 6. The power-bar now being used for traction force, the same rigidity is not as necessary as in the other apparatus. I find, therefore, that a No. 16 wire, not flattened in front, is of sufficient size.

The other, or lever-bar, the force of which acts in the opposite direction to prevent the occluding ends of the teeth from being drawn back, should be as large as No. 18. It should be flattened in the same manner described for the power-bar. The upper ends of the upright bars are grooved on their anterior surfaces to form a rest for the power-bar, while a shoulder is filed on the posterior surface of the lower ends, which forms a slot, when in place, for the flattened lever-bar to rest.

It being understood with this apparatus that the power-bar nuts work at the posterior ends of the tubes, while those of the lever-bar work at the anterior ends. Proper provisions for this arrangement should be made when constructing the anchorage appliances.

(*Discussion December No.*)

## Figures and Tables

**Case 1. f1:**
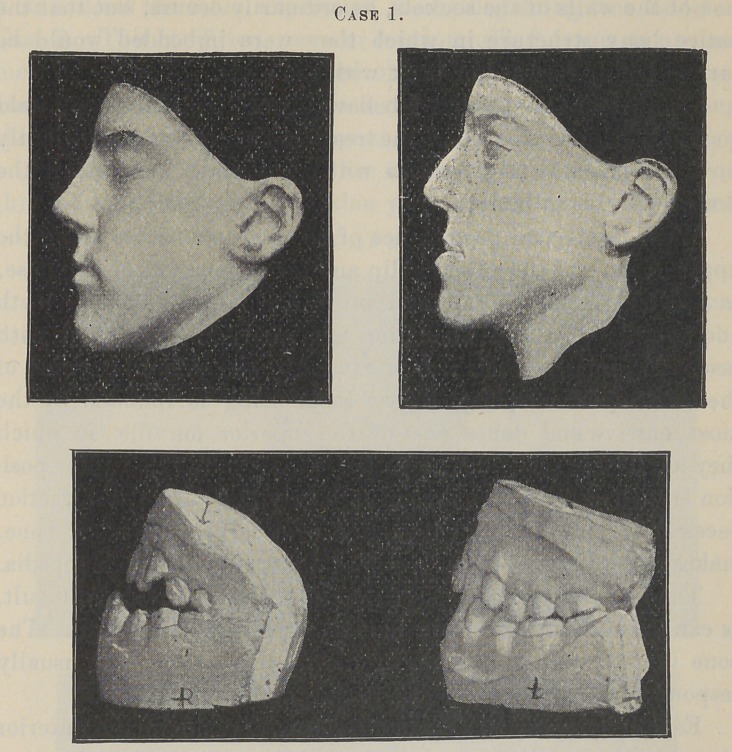


**Case 2. f2:**
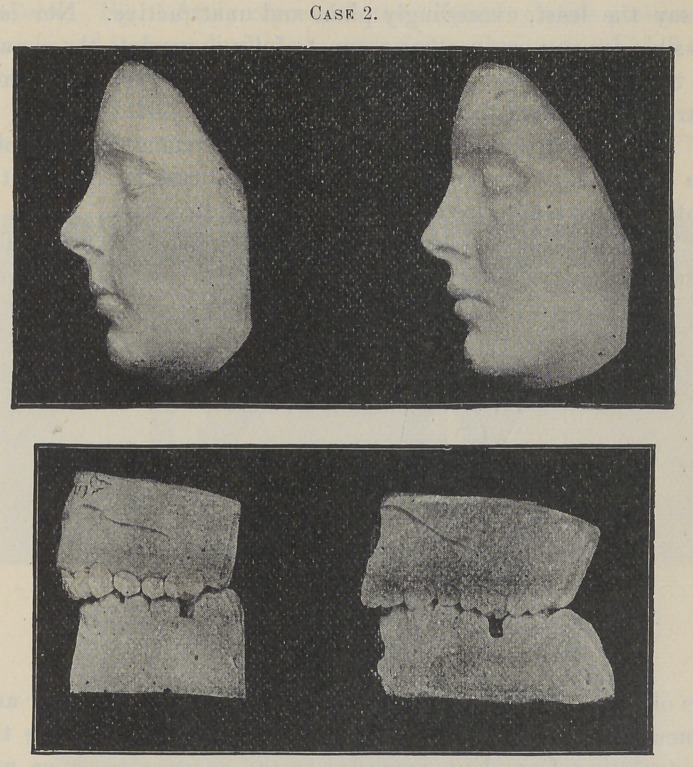


**Fig. 1. f3:**
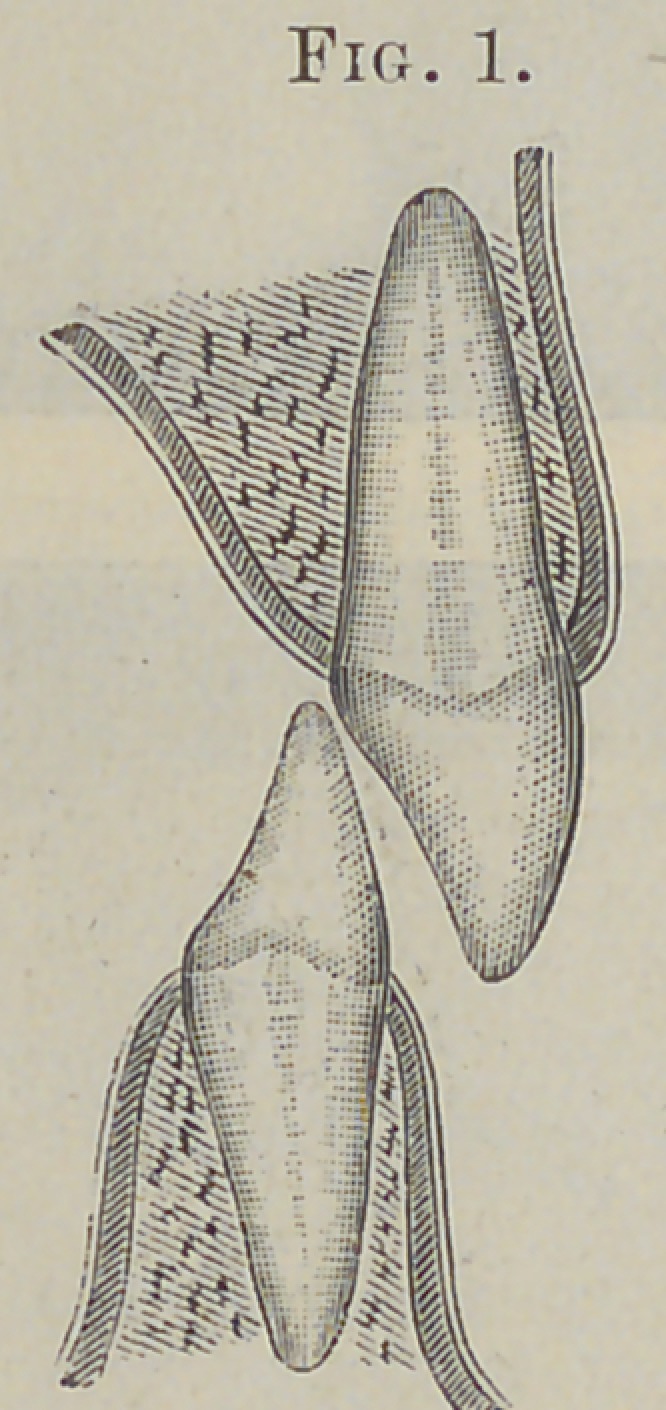


**Fig. 2. f4:**
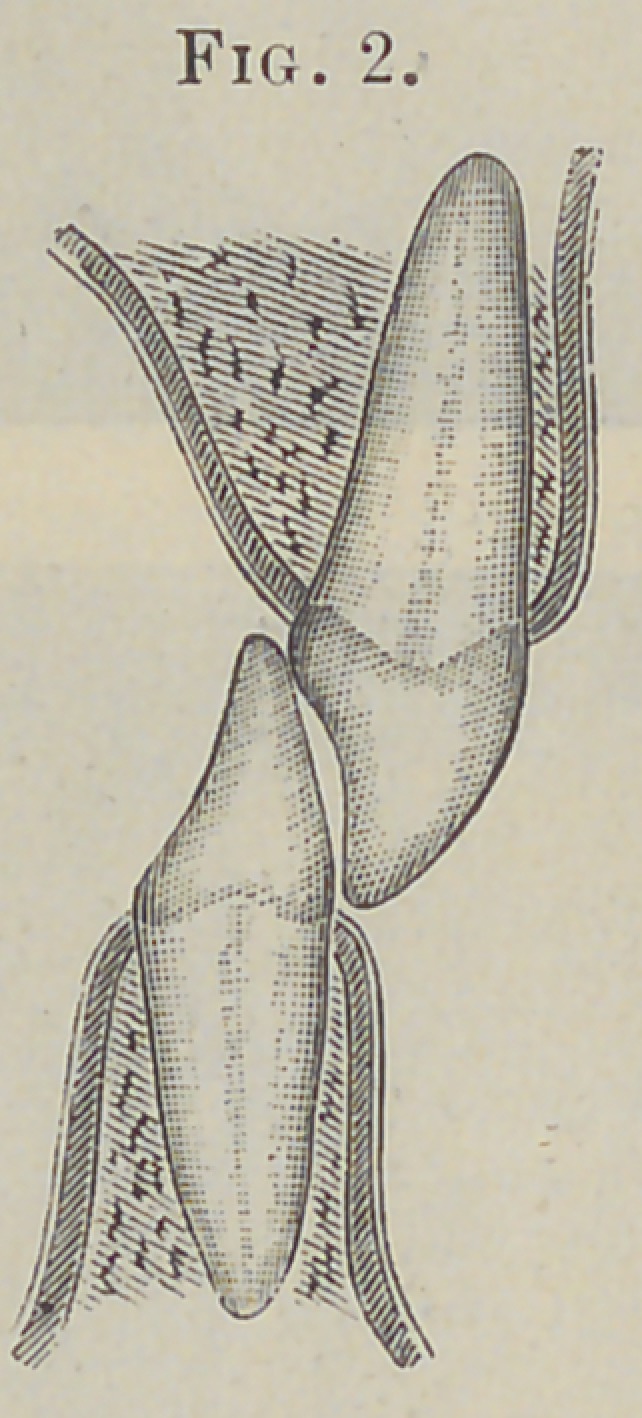


**Case 3. f5:**
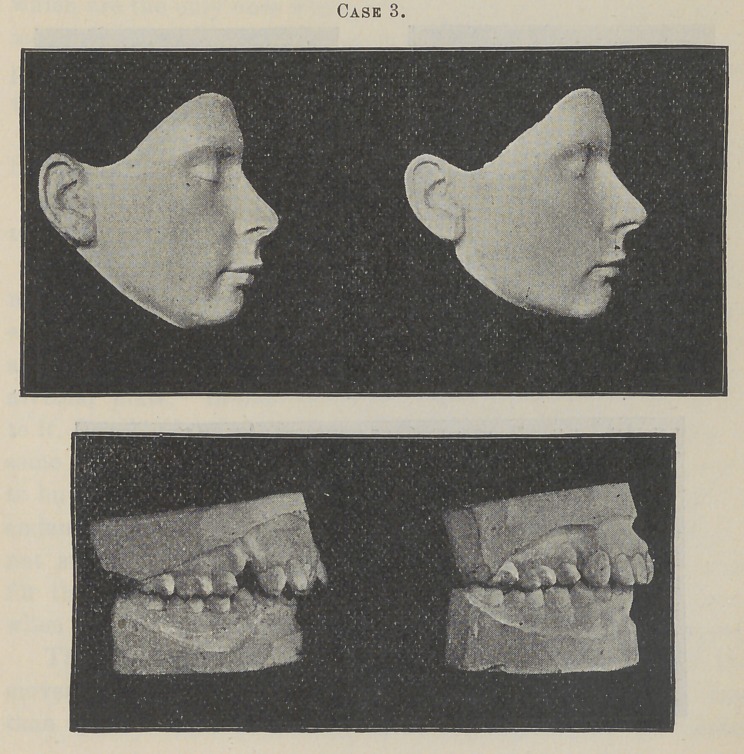


**Case 4. f6:**
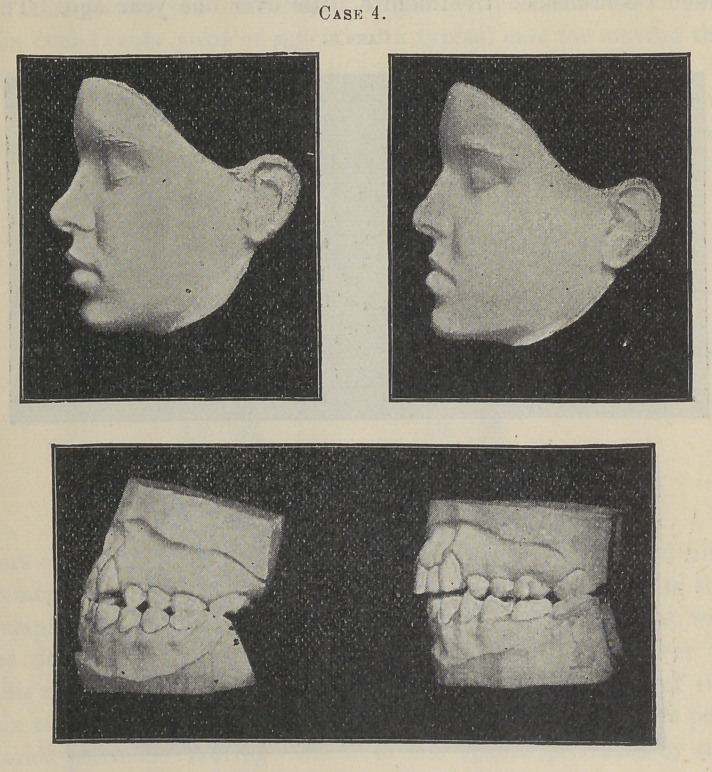


**Case 5. f7:**
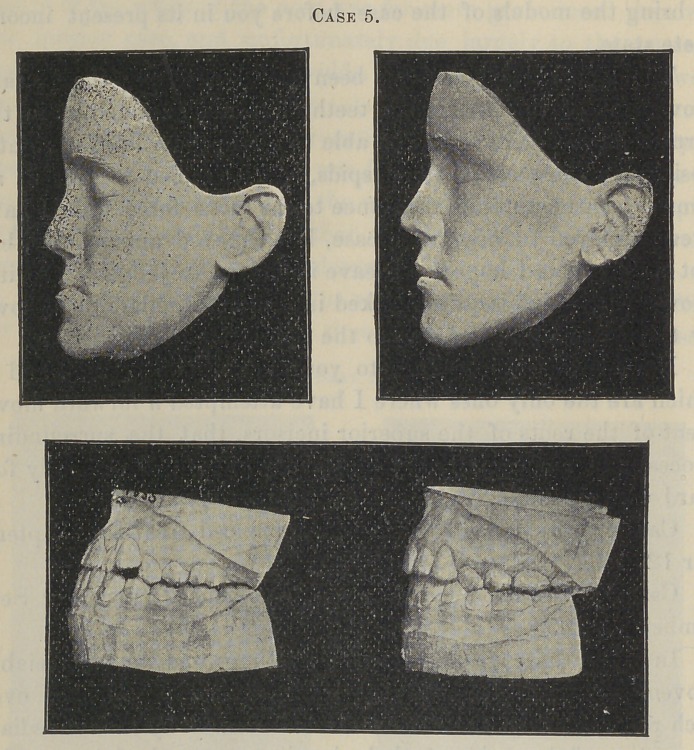


**Case 6. f8:**
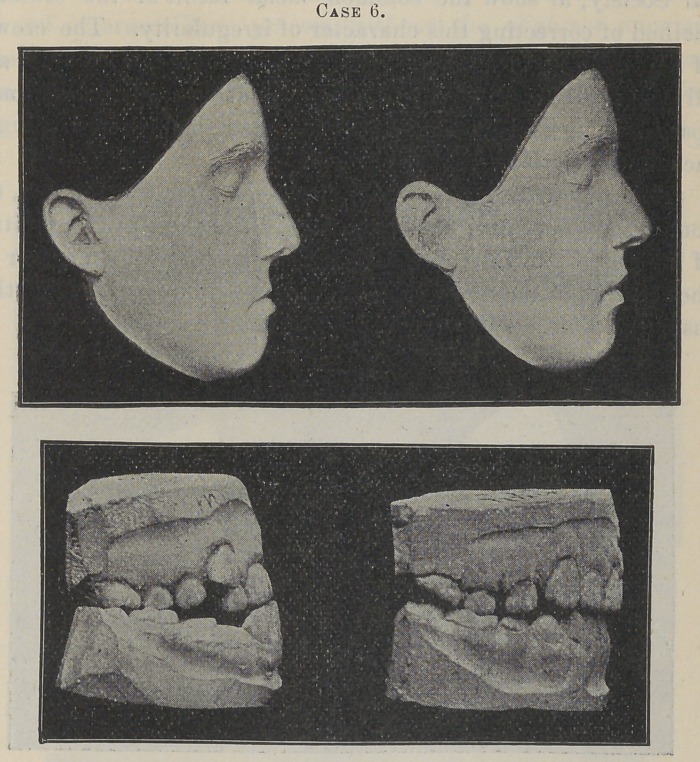


**Case 7. f9:**
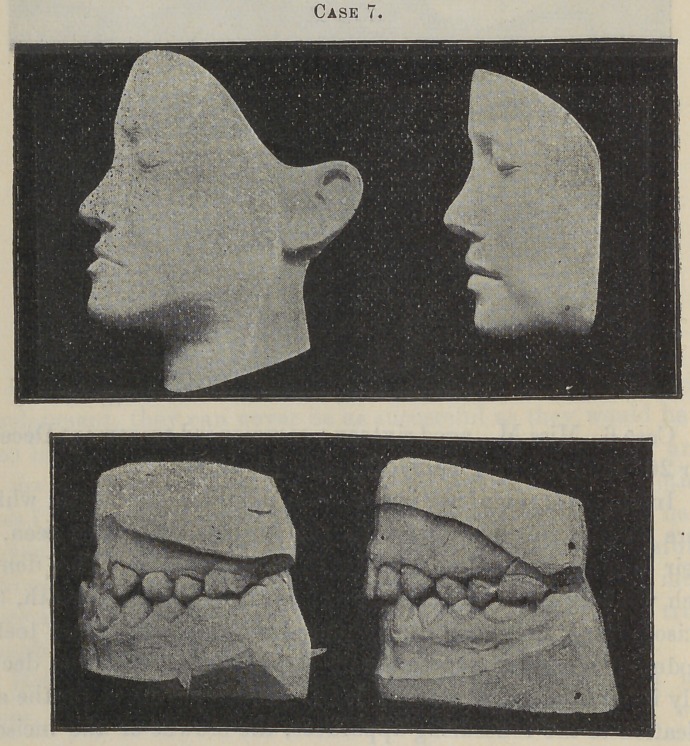


**Case 8. f10:**
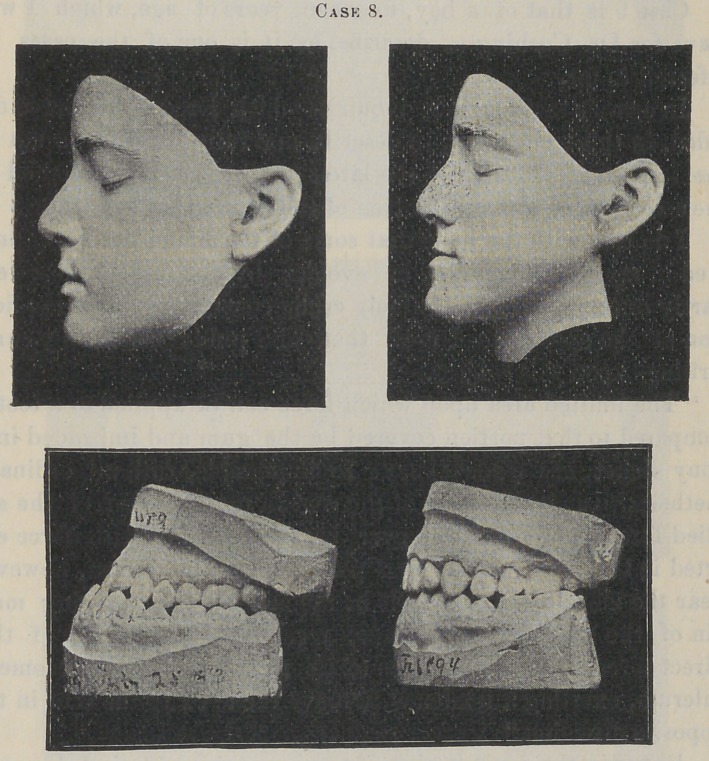


**Case 9. f11:**
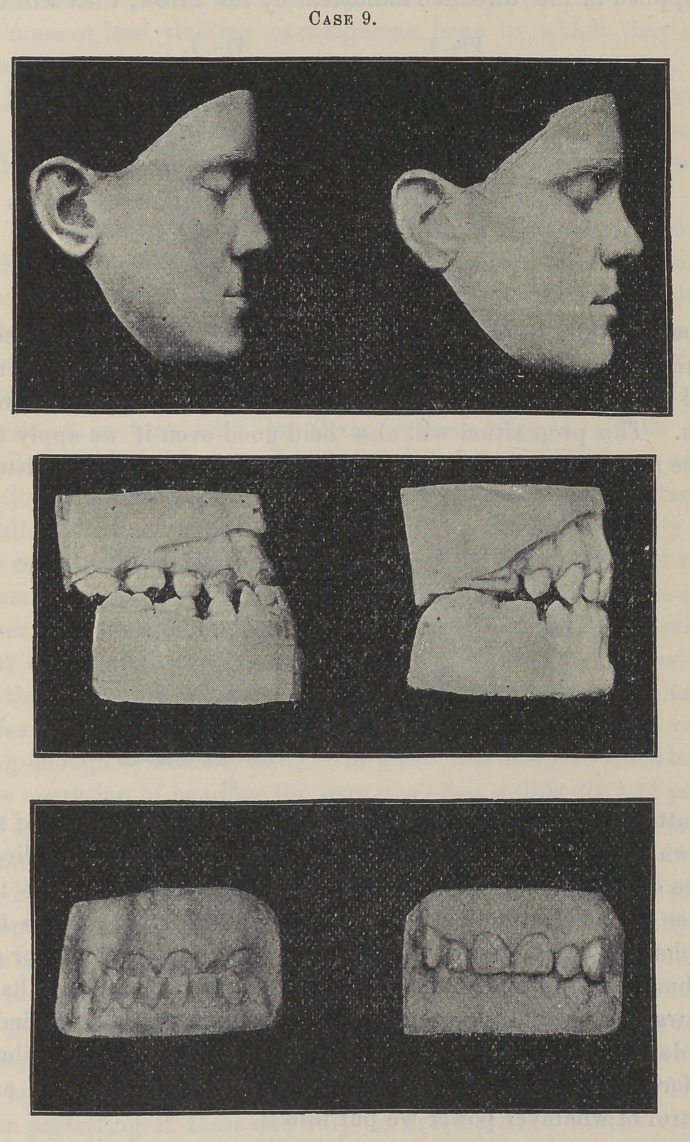


**Fig. 3. f12:**
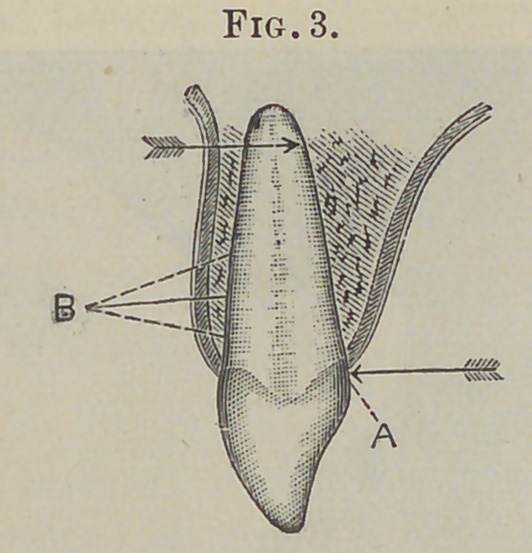


**Fig. 4. f13:**
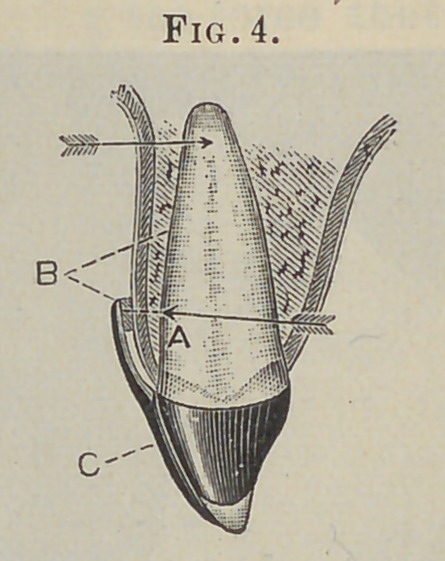


**Fig. 5. f14:**
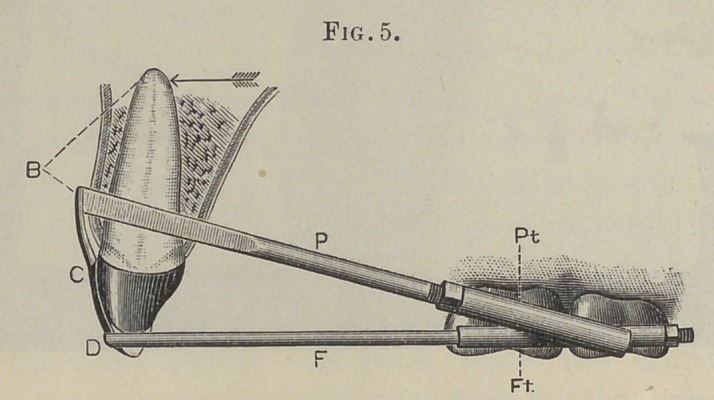


**Fig. 6. f15:**